# The M1 Paradox: Pro-Tumorigenic Effect of Macrophage Cytotoxicity in Prostate Cancer

**DOI:** 10.3390/ijms27083655

**Published:** 2026-04-20

**Authors:** Olga V. Kovaleva, Vasiliy V. Sinyov, Madina A. Rashidova, Olga S. Malashenko, Polina A. Podlesnaya, Pavel B. Kopnin, Maria V. Vasileva, Alexander S. Balkin, Andrey Plotnikov, Alexei Gratchev

**Affiliations:** 1 Institute of Experimental Oncology and Carcinogenesis, N.N. Blokhin National Medical Research Center of Oncology, 115522 Moscow, Russia; ovkovaleva@gmail.com (O.V.K.); v.sinyov@ronc.ru (V.V.S.); m.rashidova@ronc.ru (M.A.R.); o.malashenko@ronc.ru (O.S.M.); p.podlesnaya@ronc.ru (P.A.P.); pbkopnin@mail.ru (P.B.K.); mvnovikova94@mail.ru (M.V.V.); 2 Institute for Cellular and Intracellular Symbiosis, Orenburg Federal Research Center, Ural Branch of Russian Academy of Sciences, 460000 Orenburg, Russia; balkinas@yandex.ru (A.S.B.); protoz@mail.ru (A.P.); 3 Center for Bio- and Medical Technologies, Skolkovo Institute of Science and Technology, 121205 Moscow, Russia

**Keywords:** macrophage, prostate cancer, tumor microenvironment, tumor-associated macrophages, macrophage plasticity, cytotoxicity

## Abstract

Macrophages are highly plastic cells of the tumor microenvironment, and although classically activated M1 macrophages are generally regarded as anti-tumor effectors, their prolonged cytotoxic activity may also promote tumor adaptation. In this study, we investigated whether sustained exposure of prostate cancer cells to cytotoxic M1-like macrophages results in the selection of tumor cell populations with enhanced malignant properties. Macrophage-resistant derivatives of the human prostate cancer cell lines PC3 and DU145 were generated by repeated co-culture with cytotoxic THP-1-derived macrophages and characterized *in vitro* and *in vivo*. Macrophage-selected tumor cells showed increased proliferative activity and enhanced clonogenic survival. *In vivo*, these cells formed larger xenograft tumors with more aggressive histopathological features. At the same time, their migratory activity was not significantly increased, although they displayed partial epithelial–mesenchymal transition-like changes, including increased vimentin expression without a marked loss of epithelial markers. Transcriptomic profiling revealed coordinated inflammatory and adaptive reprogramming, characterized by the enrichment of cytokine- and immune-response pathways together with the suppression of metabolic and differentiation-associated programs. These changes were accompanied by the selective activation of p38 MAPK signaling, while sensitivity to paclitaxel remained unchanged. Taken together, the results indicate that macrophage cytotoxicity may act as a selective pressure promoting the emergence of inflammation-adapted tumor cell variants with increased malignant potential, supporting re-evaluation of the role of M1-like macrophages in tumor progression.

## 1. Introduction

Solid tumors represent highly complex and dynamic systems composed not only of malignant cells but also of recruited mesenchymal, epithelial, and immune cell populations that form the tumor microenvironment (TME). Continuous communication between tumor cells and stromal components shapes tumor development, influencing proliferation, invasion, immune escape, and therapeutic response. Among the cellular components of the TME, immune cells—including macrophages, neutrophils, and lymphocytes—represent key regulators of tumor-associated inflammation and tissue remodeling [1].

Macrophages represent one of the dominant immune cell populations infiltrating solid tumors. These cells exhibit remarkable functional plasticity and can dynamically adapt their phenotype and functions in response to environmental signals. Similar to T-cell functional polarization, macrophages have historically been classified within a simplified dichotomous framework into classically activated, pro-inflammatory M1 macrophages and alternatively activated, anti-inflammatory M2 macrophages. M1 macrophages are characterized by cytotoxic activity, production of inflammatory cytokines, and the ability to eliminate infected or transformed cells, whereas M2 macrophages are associated with tissue repair, immune suppression, and resolution of inflammation [2,3].

Within the context of cancer, tumor-associated macrophages (TAMs) have traditionally been viewed predominantly as M2-like cells, and numerous clinical studies have demonstrated a strong association between M2 macrophage infiltration and poor prognosis across multiple solid tumor types [4,5,6,7]. In contrast, M1 macrophages have long been considered protective effectors capable of mediating anti-tumor immunity through direct cytotoxicity and activation of adaptive immune responses [8,9]. This paradigm has strongly influenced therapeutic strategies aimed at reprogramming macrophages toward an M1 phenotype as a means of enhancing anti-tumor immunity.

Recent work has also shown that M1-oriented macrophage-based approaches may have therapeutic value in selected settings. For example, macrophage-engineered vesicles derived from M1 macrophages have demonstrated anti-tumor activity in ovarian cancer models and were able to promote repolarization toward an M1-like macrophage state [10,11]. In addition, cisplatin-loaded M1 macrophage-derived vesicles have shown anti-cancer activity in osteosarcoma, supporting the use of M1-based platforms for both drug delivery and immune modulation [12]. These findings indicate that M1-like macrophages can be therapeutically beneficial in some contexts, which further emphasizes that the biological consequences of macrophage activation are likely to be highly context dependent.

However, despite substantial experimental efforts, macrophage-targeted therapies designed to reprogram macrophages and promote their cytotoxic or pro-inflammatory polarization have not yet delivered consistent, broadly reproducible clinical benefit across tumor types, and their translation has been uneven. Notable exceptions in defined clinical settings suggest that pro-inflammatory myeloid activation can be effective, but these successes have not straightforwardly generalized to most solid tumors [13]. This discrepancy raises an important and largely unresolved question: why does enforced activation of macrophage cytotoxicity so often fail to translate into robust therapeutic benefit? One possible explanation is that the functional consequences of prolonged pro-inflammatory macrophage activity within tumors remain incompletely understood.

Tumors are highly plastic systems that evolve under continuous selective pressure imposed by the microenvironment, including immune cells. Under these conditions, activation of macrophage cytotoxicity may not necessarily lead to complete elimination of tumor cells. Instead, prolonged exposure to inflammatory macrophages may favor the survival of tumor cell variants capable of resisting cytotoxic attack and adapting to inflammatory stress. Such a process could ultimately contribute to tumor progression by promoting the emergence of more aggressive cell populations. In this context, the concept of the “M1 paradox” may reflect not a direct tumor-promoting function of inflammatory macrophages, but rather their ability to act as a selective force shaping tumor evolution.

The aim of the present study was to investigate the consequences of sustained exposure of prostate cancer cells to cytotoxic M1-like macrophages and to determine whether macrophage-driven selection gives rise to tumor cell populations with enhanced malignant properties.

## 2. Results

**Figure 1:**
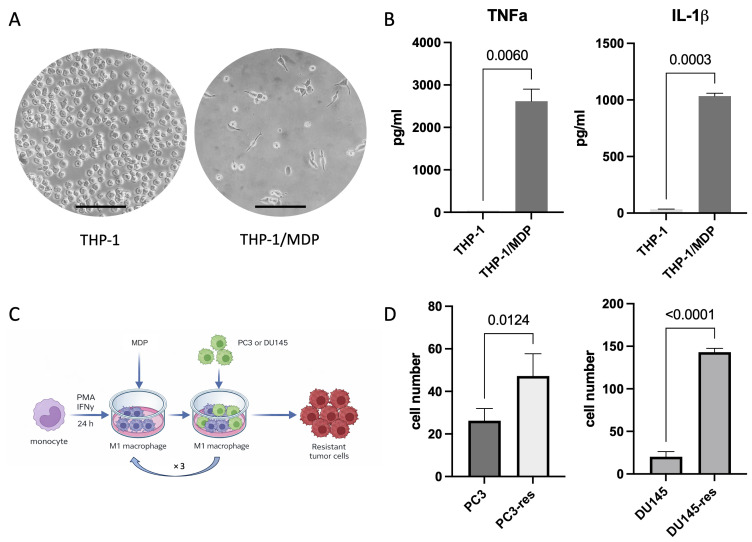
Generation and characterization of cytotoxic THP-1-derived M1-like macrophages and selection of macrophage-resistant tumor cells. (**A**) Morphological changes in THP-1 cells following differentiation and stimulation with PMA, IFNγ, and MDP, resulting in acquisition of an adherent M1-like phenotype. Scale bar corresponds to 100 μm. (**B**) Production of TNF-α and IL-1β by THP-1-derived macrophages following MDP stimulation. Data represent mean ± SD, *n* = 3. (**C**) Schematic representation of the experimental design used for repeated co-culture of prostate cancer cells with cytotoxic macrophages and selection of resistant tumor cell derivatives. (**D**) Comparative analysis of the sensitivity of parental and macrophage-selected tumor cell lines to macrophage cytotoxicity. Data represent mean ± SD, *n* = 5.

To investigate the impact of macrophage cytotoxicity on tumor cell behavior, we used a previously established experimental model that allows selection of tumor cells resistant to macrophage cytotoxicity [14]. Through repeated co-culture with cytotoxic THP-1-derived macrophages, resistant derivatives of the prostate cancer cell lines PC3 and DU145 were generated and used for subsequent analyses. To obtain cytotoxic macrophages, THP-1 cells were first differentiated and then stimulated with MDP for 24 h. The resulting cells were adherent and displayed morphology characteristic of M1-like macrophages (Figure 1A). Acquisition of a cytotoxic pro-inflammatory phenotype was further confirmed by analysis of TNF-α and IL-1β production (Figure 1B). MDP-stimulated macrophages produced high levels of both cytokines, consistent with their inflammatory activation state.

To generate tumor cell populations resistant to macrophage cytotoxicity, PC3 and DU145 cells were co-cultured with THP-1-derived cytotoxic macrophages (Figure 1C). Tumor cell resistance was then evaluated using the same co-culture system, with the corresponding parental cell lines serving as controls. This analysis showed that repeated co-cultivation resulted in the generation of prostate cancer cell derivatives with increased resistance to macrophage cytotoxicity. The selected sublines were designated PC3-res and DU145-res (Figure 1D).

### 2.1. Proliferative Activity and Clonogenic Potential of Resistant Tumor Cells

**Figure 2:**
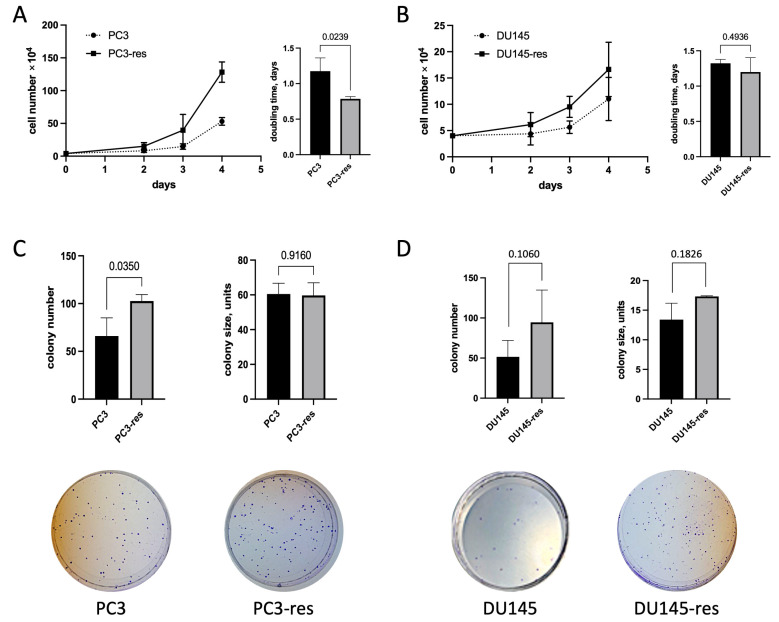
Proliferative activity and clonogenic potential of macrophage-selected prostate cancer cells. Growth kinetics and population doubling time of parental and macrophage-selected PC3 (**A**) and DU145 (**B**) cells. Clonogenic assay showing colony-forming ability of parental and PC3-res (**C**) and DU145-res (**D**) cells under low-density culture conditions. Representative images and quantitative analysis of colony number are shown. Data represent mean ± SD, *n* = 4.

Increased proliferative capacity is one of the key features of malignant cells. Analysis of growth kinetics showed that both PC3-res and DU145-res cells proliferated more rapidly than their corresponding parental cell lines (Figure 2). In particular, the population doubling time of PC3-res cells was approximately 18 h, whereas that of parental PC3 cells exceeded 24 h; this difference was statistically significant (*p* = 0.0239; Figure 2A). DU145-res cells also displayed a higher proliferation rate than parental DU145 cells, although this difference did not reach statistical significance (Figure 2B).

Another important feature reflecting malignant potential *in vitro* is the ability of tumor cells to form colonies under conditions of low cell density and limited availability of growth-supporting signals. To assess this property, clonogenic assays were performed. The macrophage-selected cells formed a greater number of colonies than the corresponding parental cells, whereas the average colony size remained largely unchanged. PC3-res cells formed nearly twice as many colonies as PC3 cells (Figure 2C). A similar tendency was observed for DU145-res cells compared with DU145 cells, although the difference did not reach statistical significance (Figure 2D). These findings indicate that acquisition of resistance to macrophage cytotoxicity is associated with enhanced clonogenic survival of tumor cells.

### 2.2. Migration Capacity Remains Unchanged in Macrophage-Selected Tumor Cells

**Figure 3:**
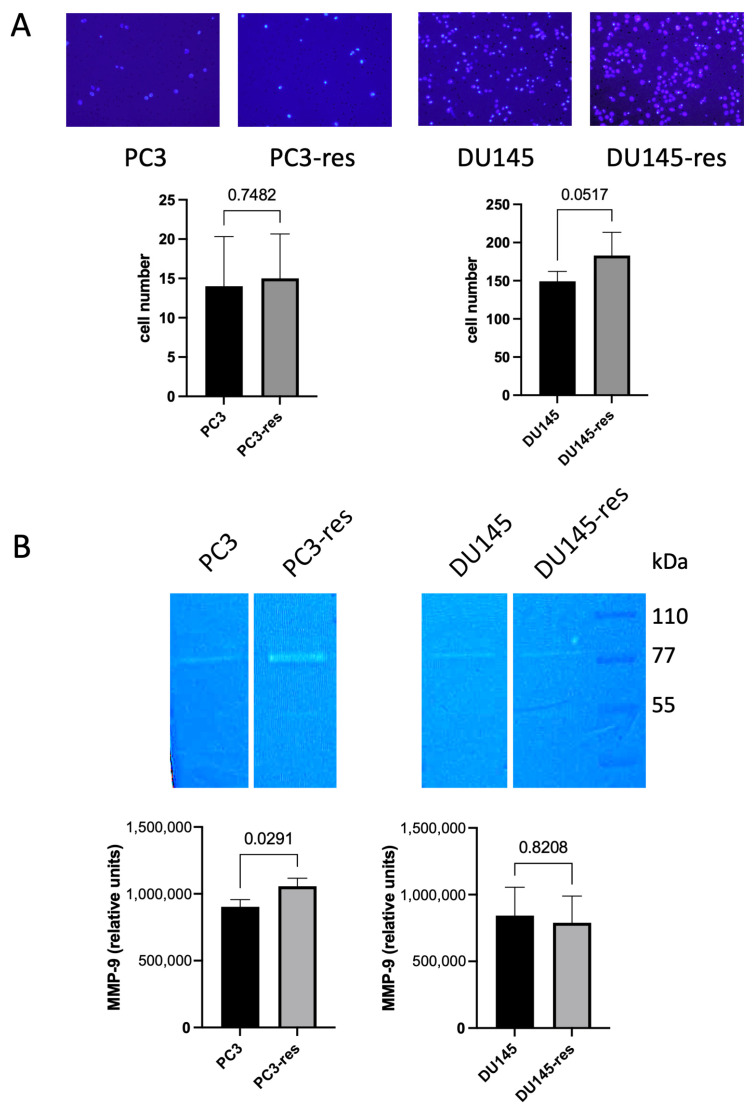
Migration capacity and gelatinase activity of macrophage-selected prostate cancer cells. (**A**) Transwell migration assay of parental and macrophage-selected PC3 and DU145 cells. Representative microphotographs of migrated cells (original magnification, ×10) and their quantitative analysis are shown. Data represent mean ± SD, *n* = 5. (**B**) Gelatin zymography of conditioned media from parental and macrophage-selected PC3 and DU145 cells demonstrating gelatinase activity. Samples were normalized to cell number prior to electrophoresis. Data represent mean ± SD, *n* = 3.

Transwell migration assays were performed to determine whether acquisition of resistance to macrophage cytotoxicity was accompanied by changes in migratory capacity. The results showed that PC3-res cells did not exhibit increased migration compared with parental PC3 cells (Figure 3A). DU145-res cells displayed a tendency toward higher migratory activity than parental DU145 cells; however, this difference did not reach statistical significance (Figure 3A). These findings indicate that macrophage-driven selection does not substantially alter the migratory properties of prostate cancer cells under the conditions used in this study.

To further determine whether macrophage-driven selection influences invasion-associated properties of tumor cells, gelatinase activity was assessed by gelatin zymography. Matrix metalloproteinases (MMPs), particularly the gelatinases MMP-2 and MMP-9, are important mediators of extracellular matrix remodeling and are commonly associated with tumor progression. Conditioned media collected from parental and macrophage-selected PC3 and DU145 cells were therefore analyzed for gelatinase activity.

Zymographic analysis showed that all examined cell lines predominantly secreted MMP-9, whereas MMP-2 activity was not detected. Acquisition of resistance to macrophage cytotoxicity was associated with increased MMP-9 activity in PC3-res cells, while no substantial changes were observed in DU145-res cells (Figure 3B). Thus, although macrophage-driven selection did not significantly affect migratory activity under the conditions used, it was accompanied by increased secretion of MMP-9 in PC3-res cells, indicating partial changes in invasion-associated properties.

### 2.3. Epithelial–Mesenchymal Transition Markers in Macrophage-Selected Tumor Cells

Since macrophage-driven selection resulted in increased proliferative and clonogenic activity, while only limited effects on migration were observed, we next asked whether these phenotypic changes were accompanied by alterations associated with epithelial–mesenchymal transition (EMT). EMT is widely recognized as a process contributing not only to tumor invasion and dissemination, but also to cellular plasticity and adaptation to stressful microenvironmental conditions. In this context, even partial EMT-like changes may reflect acquisition of a more adaptable and aggressive tumor cell state. Therefore, to determine whether macrophage-selected tumor cells had undergone phenotypic reprogramming consistent with EMT-related plasticity, the expression of key epithelial and mesenchymal markers was analyzed in parental prostate cancer cells and their macrophage-selected derivatives by immunocytochemistry.

**Figure 4:**
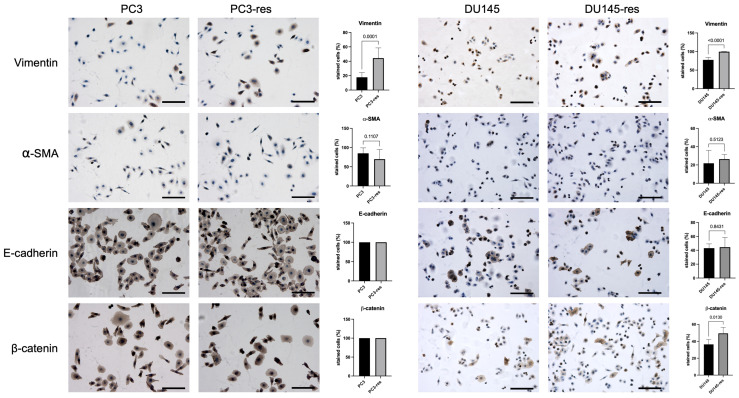
Immunocytochemical analysis of epithelial and mesenchymal marker expression in macrophage-selected prostate cancer cells. Representative immunocytochemical staining of parental and macrophage-selected PC3 (**left panel**) and DU145 (**right panel**) cells for the epithelial markers E-cadherin and β-catenin and the mesenchymal markers smooth muscle actin (SMA) and vimentin, together with quantitative analysis of the percentage of positive cells. Staining was performed under identical experimental conditions, and representative microscopic fields are shown. Cells with visible brown DAB staining above background were scored as positive, irrespective of staining intensity. For E-cadherin and β-catenin in the PC3 model, all counted cells were positive in both parental and macrophage-selected groups (100%), and therefore no variability is visible in the corresponding graphs. Data represent mean ± SD, *n* = 5. Scale bar corresponds to 100 μm.

Immunocytochemical analysis revealed changes in the expression of EMT-associated markers in macrophage-selected tumor cell sublines compared with the corresponding parental cells (Figure 4). In both PC3-res and DU145-res cells, expression of the mesenchymal marker vimentin was increased relative to the respective control cells. In contrast, the expression levels of the epithelial markers E-cadherin and β-catenin did not show substantial differences between parental and macrophage-selected cells. These findings indicate that macrophage-driven selection is associated with acquisition of certain mesenchymal features without evidence of a complete EMT program.

### 2.4. Macrophage-Selected Tumor Cells Exhibit Enhanced Tumorigenic Potential In Vivo

Since acquisition of resistance to macrophage cytotoxicity was associated with altered tumor cell properties *in vitro*, we next examined whether these changes were also reflected *in vivo*. To address this question, xenograft experiments were performed to compare the tumor-forming ability of parental and macrophage-selected prostate cancer cells.

**Figure 5:**
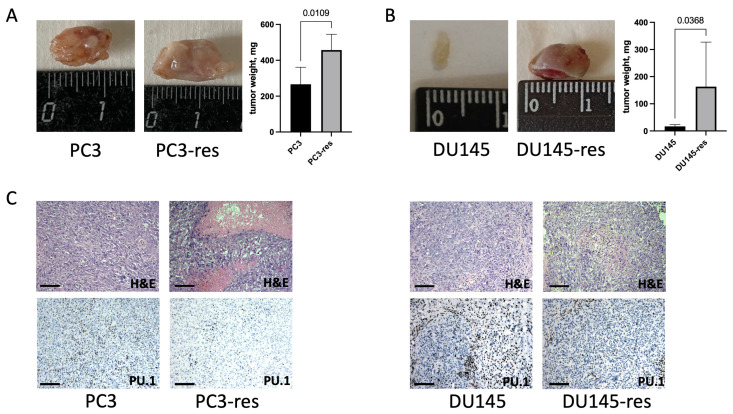
Enhanced tumorigenic potential and histological features of macrophage-selected prostate cancer cells *in vivo*. Representative images of subcutaneous xenograft tumors formed by parental and macrophage-selected prostate cancer cells and final tumor weight measured at the experimental endpoint for PC3 (**A**) and DU145 (**B**) cells. Data represent mean ± SD, *n* = 5. Representative histological sections of xenograft tumors stained with hematoxylin and eosin (H&E), together with immunohistochemical staining for PU.1. H&E staining was used to assess tumor morphology, PU.1 immunostaining was used to evaluate the presence of myeloid cells within the tumor tissue (**C**). Scale bar corresponds to 100 μm.

*In vivo* experiments demonstrated that tumor cells resistant to macrophage cytotoxicity exhibited enhanced malignant properties, as reflected by the accelerated growth of subcutaneous xenografts compared with tumors formed by the corresponding parental cell lines (Figure 5A,B). Histopathological analysis further showed that tumors derived from macrophage-selected cells were characterized by larger size, more frequent necrotic areas, perineural invasion, and increased vascularization (Figure 5C). Together, these findings indicate that selection under macrophage cytotoxicity gives rise to tumor cell populations with increased tumorigenic potential *in vivo*.

Notably, tumors derived from macrophage-selected cells contained fewer PU.1-positive cells than control tumors (Figure 5C), indicating changes in the cellular composition of the tumor microenvironment. This observation suggests that selection under macrophage cytotoxicity may influence not only intrinsic properties of tumor cells, but also their interactions with stromal and immune components *in vivo*. Thus, acquisition of resistance to macrophage cytotoxicity appears to be associated with broader alterations affecting tumor–microenvironment relationships.

### 2.5. Evaluation of Tumor Cell Sensitivity to Paclitaxel

Since macrophage-driven selection was associated with the acquisition of a more aggressive and stress-adapted phenotype, we next examined whether these changes were accompanied by altered sensitivity to chemotherapy. To address this question, the response of parental PC3 and DU145 cells and their macrophage-selected derivatives to paclitaxel was assessed by MTT assay. Sensitivity to the drug was evaluated by comparing the IC50 values obtained for each parental cell line and its corresponding macrophage-selected subline.

The analysis showed that macrophage-selected tumor cells did not display a significant change in paclitaxel sensitivity. In the PC3 model, the IC50 increased slightly from 3.59 ± 0.52 nM in parental PC3 cells to 4.35 ± 0.46 nM in PC3-res cells; however, this difference was not statistically significant (*p* = 0.336). Similarly, in the DU145 model, the IC50 values were 2.70 ± 0.77 nM for parental DU145 cells and 2.99 ± 0.73 nM for DU145-res cells, with no significant difference between the groups (*p* = 0.809). Thus, although macrophage-driven selection was associated with the acquisition of several more aggressive features and partial EMT-like characteristics, these changes were not accompanied by a measurable alteration in paclitaxel sensitivity under the conditions used in this study.

### 2.6. Transcriptome Analysis

Since macrophage-driven selection was associated with increased proliferative activity, enhanced clonogenicity, partial EMT-like changes, and greater tumorigenic potential *in vivo*, we next sought to determine whether these phenotypic alterations were accompanied by broader transcriptional reprogramming. For this purpose, transcriptomic profiling was performed using parental PC3 cells and their macrophage-selected derivatives, PC3-res, as a representative model for molecular characterization. This analysis was undertaken to identify gene expression changes associated with adaptation to macrophage cytotoxicity and to gain insight into the molecular programs potentially underlying the altered biological properties of the selected tumor cells.

Sequencing of cDNA libraries using Illumina HiSeq 2000 platform generated a total of 267,218,048 single-end reads. After the removal of adapter sequences and rDNA reads, an average of 29.08 ± 4.54 million reads per library was retained. Alignment to the human reference genome (GRCh38.p12) demonstrated high mapping efficiency, with 97.72–97.92% of reads successfully aligned, and the majority of which mapped uniquely (>88%).

Differential expression analysis revealed 344 genes with significantly altered expression in PC3-res cells compared to the original PC3 cells (|log_2_ fold change| > 1, FDR-adjusted *p* < 0.05). Among these, 148 genes were upregulated, whereas 196 genes were downregulated. Gene ontology enrichment analysis revealed activation of pathways associated with cytokine response, immune regulation, cell communication, and adhesion in PC3-res cells. In contrast, downregulated genes were predominantly related to responses to jasmonic acid, as well as polyketide, alcohol, and steroid metabolic processes.

**Figure 6:**
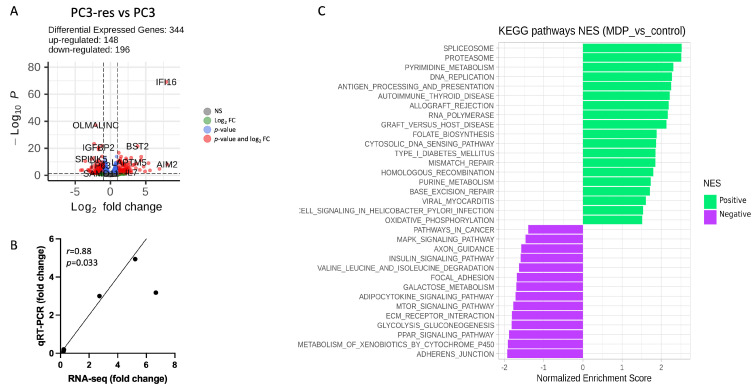
Transcriptomic characterization of macrophage-selected PC3 cells. (**A**) Volcano plot showing differentially expressed genes in PC3-res cells compared with parental PC3 cells. Vertical dashed lines indicate the log_2_ fold-change thresholds, and the horizontal dashed line indicates the statistical significance threshold. Genes meeting both criteria were considered differentially expressed. (**B**) Correlation between gene expression changes determined by RNA-seq and qRT-PCR for selected genes. Expression values are presented relative to parental control cells. (**C**) Gene set enrichment analysis of KEGG pathways in macrophage-selected PC3-res cells compared with parental PC3 cells. Bars indicate normalized enrichment score (NES); positive NES values represent pathways enriched among upregulated genes, whereas negative NES values represent pathways enriched among downregulated genes.

To further validate the RNA-seq findings, quantitative RT-PCR analysis was performed for six randomly selected genes using the same RNA samples. The expression patterns detected by qRT-PCR correlated strongly with RNA-seq results (R = 0.82, *p* = 0.0058), confirming the reliability of the transcriptomic data (Figure 6).

Gene set enrichment analysis revealed that KEGG pathways related to NOD-like receptor signaling, spliceosome, ribosome biogenesis in eukaryotes, RNA transport, cytosolic DNA-sensing pathway, and the proteasome were significantly enriched in the PC3-res libraries. In contrast, pathways associated with metabolism of xenobiotics by cytochrome P450, ECM–receptor interaction, PPAR signaling pathway, chemical carcinogenesis, AMPK signaling pathway, steroid hormone biosynthesis, and insulin resistance were significantly enriched in control libraries (Table 1).

Pathway enrichment analysis suggested that macrophage-driven selection primarily induced inflammatory stress adaptation and transcriptional reprogramming rather than activation of classical invasion-associated programs.

Transcriptomic analysis followed by the functional categorization of differentially expressed genes revealed coordinated changes affecting several major biological programs in macrophage-selected tumor cells (Table 2). Genes associated with innate immune signaling and cytokine responsiveness were predominantly upregulated, indicating activation of inflammation-related pathways within tumor cells. In parallel, the increased expression of genes involved in immune interaction and antigen presentation suggested enhanced responsiveness to signals originating from the inflammatory microenvironment. Additionally, transcriptional changes were observed in gene groups related to cellular plasticity and interaction with the tumor microenvironment, consistent with adaptive remodeling of tumor cell properties under macrophage-mediated selective pressure. In contrast, genes associated with metabolic regulation and epithelial differentiation were generally downregulated, indicating a shift from specialized metabolic functions toward an adaptive stress-associated state.

Overall, these transcriptional changes indicate that macrophage-driven selection induces the coordinated inflammatory and adaptive reprogramming of tumor cells. Rather than activating a classical migration- or invasion-associated transcriptional program, selection under macrophage cytotoxicity appears to promote an inflammation-adapted cellular state characterized by enhanced stress responsiveness, altered immune-related signaling, and increased capacity for survival under adverse microenvironmental conditions.

### 2.7. Activation of p38 MAPK Signaling in Macrophage-Selected Tumor Cells

**Figure 7:**
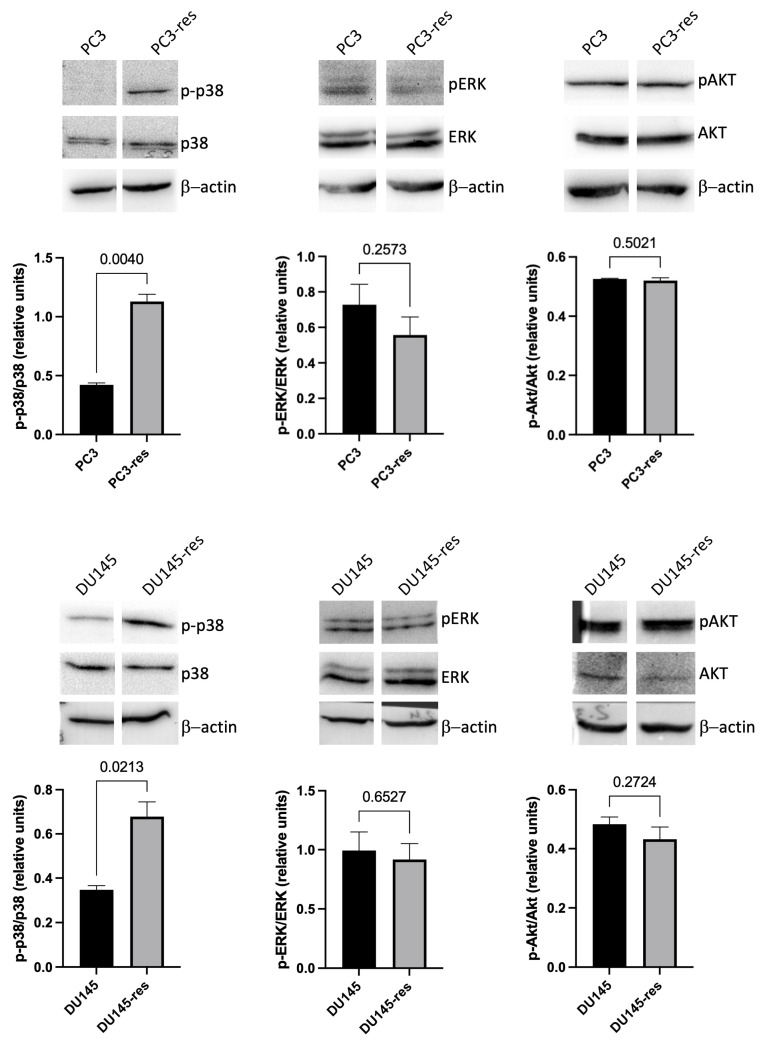
Selective activation of p38 MAPK, but not ERK1/2 or AKT, in macrophage-selected prostate cancer cells. Representative western blot analysis of phosphorylated p38 MAPK (Thr180/Tyr182), total p38, phosphorylated ERK1/2 (Thr202/Tyr204), total ERK1/2, phosphorylated AKT (Ser473), and total AKT in parental and macrophage-selected PC3 and DU145 cells. β-Actin was used as a loading control. Densitometric quantification of pathway activation was performed as the ratio of phosphorylated to total protein and is presented in relative units normalized to the corresponding parental control cells. Data are shown as mean ± SD, *n* = 3. Increased phosphorylation was observed for p38 MAPK, whereas no significant changes were detected for ERK1/2 or AKT.

Since transcriptomic analysis revealed the enrichment of cytokine and innate immune response programs together with evidence of adaptive reprogramming, we next examined whether these changes were associated with activation of stress-responsive signaling pathways. Special attention was paid to p38 MAPK, which is known to play a central role in cellular responses to inflammatory and stress-related stimuli. Immunoblot analysis demonstrated increased phosphorylation of p38 (Thr180/Tyr182) in PC3-res and DU145-res cells compared with the corresponding parental cell lines, whereas total p38 expression remained largely unchanged (Figure 7).

Taken together, these findings indicate that macrophage-driven selection is associated with activation of the p38 MAPK pathway in prostate cancer cells. In contrast, no significant changes were detected in ERK or AKT phosphorylation under the same experimental conditions (Figure 7), suggesting that the observed signaling changes are not part of a generalized activation of major growth-related pathways. Rather, these results support the idea that selection under macrophage cytotoxicity is accompanied by the preferential engagement of stress-responsive signaling mechanisms.

## 3. Discussion

The historically established M1/M2 macrophage paradigm, according to which classically activated M1 macrophages exert anti-tumor effects whereas alternatively activated M2 macrophages promote tumor progression, remains widely accepted in the field [15]. Consistent with this concept, numerous macrophage-targeted therapeutic strategies have been developed with the aim of reprogramming tumor-associated macrophages from an M2- to an M1-like phenotype as an anti-cancer approach [7,16]. However, accumulating evidence indicates that M1 macrophages do not invariably induce tumor regression and, under certain conditions, may instead contribute to tumor adaptation and progression [17]. On the basis of these observations, we hypothesized that cytotoxic M1 macrophages may promote tumor progression not directly, but by selectively favoring the survival of tumor cell subpopulations resistant to macrophage cytotoxicity. To address this question, we used a previously described experimental model that enables the selection of tumor cells under sustained exposure to cytotoxic macrophages [14]. In the present study, this approach was applied to two prostate cancer cell lines, PC3 and DU145, used as representative models. The resulting macrophage-selected sublines stably maintained their acquired properties during long-term cultivation, which made it possible to perform comprehensive functional characterization both *in vitro* and *in vivo*.

Importantly, this model was designed to address the consequences of repeated cytotoxic selection by M1-like macrophages rather than the full spectrum of later bidirectional tumor–macrophage interactions. Our data therefore support the concept that prolonged macrophage cytotoxicity can act as a selective pressure, favoring the emergence of inflammation-adapted tumor cell variants with enhanced malignant properties. At the same time, the present experiments do not establish how these selected tumor cells subsequently influence macrophage behavior in a renewed co-culture setting. Accordingly, the pro-tumorigenic effect proposed here should be understood as an indirect, selection-mediated consequence of sustained inflammatory pressure rather than as a universal direct effect of M1-like macrophages.

Both *in vitro* and *in vivo* analyses showed that prostate cancer cells that acquired resistance to macrophage cytotoxicity displayed increased proliferative capacity and enhanced tumorigenic potential. Notably, this phenotype was not accompanied by a significant increase in migratory activity. Instead, macrophage-selected tumor cells exhibited enhanced proliferation and clonogenicity without evidence of a generalized pro-invasive phenotype. Such a dissociation is compatible with a partial EMT-like state, in which tumor cells acquire selected mesenchymal and stress-adaptive features while retaining epithelial characteristics, without necessarily becoming more motile [18]. Consistent with this interpretation, transcriptomic analysis did not reveal the activation of a classical invasion-associated program.

Immunocytochemical analysis further supported this view by demonstrating selective EMT-associated alterations, most notably increased vimentin expression in the absence of detectable changes in E-cadherin expression. This phenotype is consistent with partial epithelial–mesenchymal transition (partial EMT), in which tumor cells acquire certain mesenchymal features while retaining epithelial characteristics. Increasing evidence indicates that partial EMT states are associated primarily with enhanced cellular plasticity and survival rather than with overt increases in motility or invasion, thereby supporting tumor adaptation under stress conditions rather than classical metastatic behavior [19,20]. The absence of a complete EMT response in our model may reflect the fact that macrophage-mediated cytotoxic selection provides a strong inflammatory and stress-related stimulus, but not necessarily the full combination of signals typically required to drive a stable mesenchymal transition. In many tumor settings, complete EMT is promoted by additional microenvironmental inputs such as sustained TGF-β signaling, stromal fibroblast-derived factors, extracellular matrix remodeling, hypoxia, or prolonged *in vivo* selection. Thus, the phenotype observed here is more consistent with a partial EMT-like adaptive state than with a fully developed mesenchymal conversion.

Taken together, the combination of enhanced tumorigenic potential, partial EMT-like changes, and preserved migratory activity suggests that macrophage-driven selection does not simply induce a broad increase in malignant traits but rather promotes a more specific adaptive reprogramming of tumor cells. To identify signaling mechanisms that might underlie this phenotype, we next analyzed the activation of stress-responsive pathways.

In our experimental model, macrophage-driven selection of tumor cells was accompanied by an increased activation of the p38 MAPK pathway, as evidenced by enhanced phosphorylation of p38 in macrophage-selected PC3 and DU145 cells compared with their parental counterparts. This observation is consistent with the well-established role of p38 MAPK as a central mediator of cellular responses to inflammatory and stress-related stimuli. The p38 signaling pathway is activated by cytokines and environmental stress and regulates transcriptional programs controlling inflammation, survival, and cellular adaptation [21]. Increasing evidence indicates that p38 MAPK contributes to tumor progression not primarily through stimulation of proliferation, but rather through promotion of phenotypic plasticity and stress tolerance. In particular, p38 signaling has been implicated in the regulation of EMT-associated programs and mesenchymal marker expression, including induction of vimentin, in response to inflammatory or TGF-β-related signaling [22]. Taken together, these observations suggest that the activation of p38 MAPK in macrophage-selected cells is associated with engagement of a stress-adaptive signaling program that may contribute to the phenotypic reprogramming observed in our model, rather than activation of classical proliferative pathways.

The observed phenotypic and signaling changes in macrophage-selected tumor cells are in line with an emerging body of evidence indicating that pro-inflammatory macrophages may exert context-dependent pro-tumorigenic effects. For example, conditioned medium derived from M1 macrophages has been shown to enhance invasive properties of melanoma cells through the induction of matrix metalloproteinase expression [23]. Similarly, M1 macrophage-conditioned media were reported to stimulate proliferation of hepatocellular carcinoma and gastric cancer cells via activation of inflammatory signaling pathways, including NF-κB [24,25]. Moreover, recent studies have demonstrated that M1 macrophages can promote survival and invasion of oral squamous carcinoma cells through activation of ErbB2 signaling [26]. We previously showed, using a non-small cell lung cancer model, that cytotoxic macrophages promote selection of more malignant tumor cell populations, thereby contributing to tumor progression [27]. Together, these observations support the concept that inflammatory macrophages may contribute to tumor progression under specific microenvironmental conditions.

Beyond their biological significance, these findings may also have implications for therapeutic strategies aimed at macrophage reprogramming. Although shifting tumor-associated macrophages toward a more pro-inflammatory state is widely viewed as a promising anti-cancer approach, our findings suggest that persistent macrophage cytotoxicity may, under certain conditions, promote selection of tumor cell variants adapted to inflammatory stress rather than their complete elimination. Thus, macrophage reprogramming should be considered a context-dependent therapeutic strategy whose effects may vary depending on the mode and duration of macrophage activation [13,16].

From a clinical perspective, the inflammation-adapted phenotype identified in our model may also be relevant to immune-evasion pathways in prostate cancer. Although immune checkpoint regulation was not examined directly in the present study, persistent inflammatory signaling and stress adaptation in tumor cells may intersect with PD-L1-associated programs that contribute to immune escape. Recent studies indicate that PD-L1 expression in prostate cancer is associated with adverse clinicopathologic features, including higher Gleason score/Grade Group and other markers of aggressive disease, thereby providing a clinically relevant framework for interpreting inflammation-adapted tumor states [28]. In this context, our findings raise the possibility that prolonged exposure to cytotoxic macrophages may select tumor cell variants that are not only more stress tolerant, but also potentially more compatible with immune-evasive microenvironmental programs. This question will require direct investigation in future studies.

This study has several limitations. First, the experimental system was based on established prostate cancer cell lines and THP-1-derived cytotoxic M1-like macrophages; therefore, validation in more physiologically relevant models, including primary patient-derived tumor cells, will be required to assess the broader translational relevance of these findings. Second, transcriptomic profiling was performed only in the PC3/PC3-res model, and thus the identified transcriptional program should be interpreted as a model-specific example of macrophage-selected adaptation rather than as a universal signature of prostate cancer cells exposed to macrophage cytotoxicity. Third, although increased p38 phosphorylation was observed in both PC3-res and DU145-res cells, the present study did not include functional perturbation experiments to determine whether p38 MAPK activation is required for the selected phenotype. Accordingly, p38 activation should be regarded here as an associated stress-responsive signaling event rather than as a formally established mechanistic driver. In addition, although we attempted pharmacologic inhibition of p38 in a separate macrophage-resistant tumor cell model, the available inhibitors did not produce interpretable pathway suppression under our experimental conditions and therefore did not allow reliable conclusions regarding functional p38 dependence in the present study.

Overall, our findings indicate that macrophage-driven selection induces coordinated phenotypic and transcriptional reprogramming of tumor cells. The acquisition of resistance to macrophage cytotoxicity was accompanied by increased proliferative and clonogenic capacity, as well as enhanced tumor growth *in vivo*, without a corresponding increase in migratory activity. Transcriptomic analysis revealed the activation of immune- and cytokine-responsive programs together with the suppression of metabolic and differentiation-associated pathways, pointing to the adaptation of tumor cells to inflammatory stress conditions. In agreement with these changes, biochemical analysis demonstrated the selective activation of p38 MAPK signaling. Collectively, these data support the notion that exposure to cytotoxic macrophages promotes the emergence of an inflammation-adapted tumor cell state characterized by stress-responsive signaling and increased malignant potential, rather than by activation of classical proliferative or invasion-associated pathways.

## 4. Materials and Methods

### 4.1. Cells Culture and M1-Macrophage Polarization

Human monocytic THP-1 cells (ATCC, Manassas, VA, USA) and human prostate cancer cells PC3 and DU145 (ATCC, USA) were cultured in RPMI 1640 medium (PanEco, Moscow, Russia) containing 10% fetal bovine serum (Biowest, Nuaille, France) and 0.1 mg/mL streptomycin/penicillin (PanEco, Moscow, Russia). All cell lines were cultured in a humidified atmosphere with 5% CO_2_ at 37 °C. THP-1 cells were cultured at a concentration not exceeding 10^6^ cells/mL.

THP-1 cells were differentiated into macrophages by incubation with 67 nmol phorbol 12- myristate 13-acetate (PMA) (Solarbio, Beijing, China) and 0.1 µg/mL IFNγ (StemCell, Vancouver, BC, Canada) for 24 h. After that, M1 macrophages were additionally stimulated with 0.1 µg/mL muramyl dipeptide (MDP) (Sigma-Aldrich, St. Louis, MO, USA) for 24 h. The cytotoxic activity of the obtained macrophages was assessed indirectly by measuring the concentrations of the pro-inflammatory cytokines TNF-α and IL-1β in the culture medium. The analysis was performed using ELISA kits (ZAO “Vector-Best-Europe”, Moscow, Russia) according to the manufacturer’s instructions.

To obtain tumor cells resistant to the cytotoxic activity of macrophages, the prostate cancer cells PC3 and DU145 were co-cultivated with cytotoxic macrophages (Figure 1) [14]. The optimal cell ratio for co-culture was determined based on the preliminary experiment and was 10:1 (macrophages:cancer cells). For one experiment 4 × 10^5^ THP-1 cells and 4 × 10^4^ prostate cancer cells were used. Co-cultures were incubated for 5 days; afterwards the medium was replaced with the fresh one, and remaining tumor cells were further cultivated for 2 days. After three consecutive cycles of tumor cell selection, the resistance of the resulting tumor cell derivatives toward macrophage cytotoxicity was evaluated. For this purpose, the selected cells were co-cultivated with cytotoxic macrophages and compared with the parental cell lines used as controls. Cell survival was assessed by direct counting of viable tumor cells using a Gorjaev chamber (ApexLab, Moscow, Russia) after 48 h of co-culture with cytotoxic macrophages.

### 4.2. Proliferation Assay

For the analysis of proliferation dynamics, 4 × 10^4^ cells were seeded in duplicate in 6-well plates (Costar, Arlington, VA, Canada). Cell proliferation was monitored daily for 4 days. The number of cells was determined by counting in a Gorjaev chamber, with two independent counts performed for each sample.

### 4.3. Transwell Migration Assay

For the analysis of migrations 5 × 10^4^ cells were seeded into the upper chambers of Corning Costar Transwell plates (Costar, Arlington, VA, Canada) (8 μm pore size) pretreated according to the manufacturer’s instructions. After incubation for 24 h at 37 °C, the membranes were collected, and non-migrated cells were removed from the upper surface using a cotton swab. Migrated cells were fixed with 3.7% paraformaldehyde (PFA; Dia-M, Moscow, Russia) for 15 min and subsequently stained with Hoechst dye (25 μg/mL) (Dia-M, Moscow, Russia). Five independent fields per membrane were analyzed using an inverted microscope (Olympus IX-51, Tokio, Japan) at 10× magnification.

### 4.4. Clonogenicity Assay

For the clonogenic assay, 2 × 10^2^ cells were seeded onto 6 cm Petri dishes (Costar, Arlington, VA, Canada). After 7 days, the colonies formed were fixed with ethanol and stained with crystal violet. Images of the dishes were captured using a Canon 70D camera (Canon Inc, Tokyo, Japan), and the number of colonies was quantified using ImageJ software version 1.54r.

### 4.5. Tumor Xenograft Assay

For *in vivo* experiments 6–8-week-old BALB/c nude mice were randomized into experimental and control groups (*n* = 5 per group). Each animal was inoculated subcutaneously with 100 μL of tumor cells suspension containing 5 × 10^6^ cells in sterile saline solution. Tumor size was measured every day, and tumor volume was calculated using the formula: V = width^2^ × length × 0.5. All animal experiments were conducted in accordance with the ethical standards of the European Convention for the Protection of Vertebrate Animals Used for Experimental and Other Scientific Purposes. The study protocol was approved by the Biomedical Ethics Committee of the N.N. Blokhin National Medical Research Center of Oncology, Ministry of Health of the Russian Federation (№2025-1j from 18 February 2025). After 3 weeks the animals were sacrificed, and the tumors were removed, weighted and used for the preparation of FFPE tissue blocks.

### 4.6. Immunohistochemistry Assay for PU.1 Expression

Immunohistochemical staining for PU.1 was carried out as previously described [29]. Tumor tissue sections were incubated with anti-PU.1 antibody (Clone 9G7; Cell Signaling Technology, Danvers, MA, USA) at a dilution of 1:1000, followed by a HRP-conjugated goat anti-rabbit secondary antibody. The immunoreaction was developed with diaminobenzidine (DAB; Thermo Fisher Scientific, Waltham, MA, USA). PU.1-positive cells were quantified in five independent fields of view using an OLYMPUS BX53 microscope (Olympus, Tokyo, Japan) at 10× magnification.

### 4.7. Immunocytochemistry

For immunocytochemistry glass coverslips (Micromed, St. Petersburg, Russia) were placed into 12-well culture plates, and PC3 and DU145 tumor cells as well as their resistant sublines were seeded at densities sufficient to reach at least 2 × 10^4^ cells per well after 24 h. Cells were maintained under standard culture conditions. After 24 h of cultivations the culture medium was carefully removed, and cells were washed with warm phosphate-buffered saline (PBS, 37 °C). Cells were fixed with 4% PFA for 15 min, followed by three PBS washes. Permeabilization was performed using 0.1% Triton X-100 in PBS for 3 min, followed by 3 additional PBS washes. Coverslips were then incubated with primary antibodies for 30 min. Signal detection was performed using a UltraVision Quanto Detection System HRP DAB (Thermo Fisher Scientific, Waltham, MA, USA) according to the manufacturer’s instructions. After staining, coverslips were mounted in Elvanol mounting medium and allowed to set for 12 h. Stained samples were analyzed using an OLYMPUS BX53 microscope (Olympus, Tokyo, Japan) at 10× magnification. Cells showing visible brown DAB staining above background were scored as positive irrespective of staining intensity. At least 5 independent fields were quantified.

Primary antibodies used in the study included anti-E-cadherin (clone DECMA-1, 1:100 Thermo Fisher Scientific, Waltham, MA, USA), anti-smooth muscle actin (SMA), (clone 1A4, 1:400 Genemed Biosciences, South San Francisco, CA, USA), anti-β-catenin (clone CAT-5H10, 1:200 Thermo Fisher Scientific, Waltham, MA, USA), and anti-vimentin (clone VM52, 1:200 ScyTek, Logan, UT, USA).

### 4.8. Assessment of Tumor Cell Sensitivity to Paclitaxel (MTT Assay)

Tumor cells PC3 and DU145, as well as their resistant sublines generated by contact-dependent co-culture with macrophages, were seeded into 96-well culture plates at a density of 3 × 10^3^ cells per well. After 24 h, the medium was replaced with fresh medium containing paclitaxel (Sindaxel^®^, Actavis Italy S.p.A., Nerviano, Italy) at final concentrations of 1, 5, 10, 25, and 50 nM. Following 72 h of incubation, cell viability was assessed using an MTT (PanEco, Moscow, Russia) assay. Briefly, the culture medium was removed and 200 μL of MTT solution (5 mg/mL in culture medium) was added to each well, followed by incubation for 2 h at 37 °C in a CO_2_ incubator to allow formazan crystal formation. The medium was then carefully aspirated, and the crystals were dissolved in 200 μL dimethyl sulfoxide (DMSO) (PanEco, Moscow, Russia) with shaking at room temperature for 5 min (600 rpm). Absorbance was measured at 600 nm using an automated microplate analyzer (ChemWell 2910, Awareness Technology, Palm City, FL, USA).

### 4.9. Gelatinase Activity Assay (Gelatin Zymography)

To assess gelatinase activity, PC3 and DU145 tumor cells, as well as their sublines, were seeded into 6-well plates at a density of 5 × 10^5^ cells per well. The following day, the culture medium was replaced with a serum-free medium. After 24 h, the conditioned media were collected and centrifuged for 5 min at 1000× *g* to remove cellular debris. The samples were mixed with non-reducing zymography loading buffer at a 4:1 ratio and normalized to cell number prior to electrophoresis.

Proteins were separated by SDS–PAGE in 8% polyacrylamide gels containing 0.2% gelatin (Dia-M, Moscow, Russia). Following electrophoresis, the gels were incubated in renaturation buffer for 30 min at room temperature with gentle agitation and subsequently incubated in zymography developing buffer for 30 min under the same conditions. The gels were then transferred to a fresh developing buffer and incubated for 12 h at 37 °C to allow gelatinase activity. After incubation, the gels were stained with colloidal Coomassie Brilliant Blue G-250 (Dia-M, Moscow, Russia) for 6 h, destained with distilled water, and documented using a digital imaging system.

### 4.10. Western Blotting

Western blot analysis proceeded as described previously [30]. The following primary antibodies were used: anti-phospho-Thr180/Tyr182 p38 (Cell Signaling technology), anti-p38 (Cell Signaling technology), anti-phospho-Thr202/Tyr204 ERK1/2, anti-ERK1/2, anti-phospho-Ser473 Akt, and anti-Akt (Cell-Signaling Technology). After incubation with the appropriate HRP-conjugated secondary antibodies, immunoreactive bands were visualized using a Bio-Rad detection system and analyzed with Bio-Rad Imaging Software Image Lab 6.0 (Bio-Rad, Hercules, CA, USA). All results are representative of three independent experiments. Band intensities were quantified by densitometry and expressed as fold change relative to untreated control cells. Phosphorylated protein levels were normalized to total protein levels. Representative blots and densitometric data are shown from 3 independent experiments.

### 4.11. RNA Isolation and Quantitative Real-Time Polymerase Chain Reaction

Total RNA was isolated from the cells using the RNeasy Kit (QIAGEN, Hilden, Germany) in combination with the Trizol reagent (Thermo Fisher Scientific, Waltham, MA, USA) following the standard protocol. Complementary DNA (cDNA) synthesis was performed through reverse transcription using the RevertAid RT Kit (Thermo Fisher Scientific, Waltham, MA, USA). Gene expression levels were assessed by real-time PCR. Primer sequences are provided in Table 3. Amplification was carried out on a CFX96 Touch amplifier (Bio-Rad) with the following thermal cycling program: 95 °C for 5 min, followed by 40 cycles of 95 °C for 10 s, 57–60 °C for 30 s, and 72 °C for 30 s. All reactions were conducted in two independent biological replicates. Data analysis was performed using the Bio-Rad CFX Manager software. The housekeeping gene GAPDH was used as a housekeeping gene. The relative gene expression levels were calculated using the ∆∆Ct method.

### 4.12. cDNA Library Preparation, Sequencing, and Bioinformatic Analysis

cDNA library preparation and RNA sequencing were performed according to Illumina RNA-seq protocols using the Illumina HiSeq 2000 platform (Illumina Inc., San Diego, CA, USA). Approximately 30 million 57 bp long reads per sample were generated. Raw reads were processed to remove adapter sequences using bbduk from the BBTools package v39.01 [31], followed by the removal of ribosomal RNA reads using SortMeRNA v.4.3.6 [32]. Filtered reads were aligned to the human reference genome (GRCh38.p12) using HISAT2 v2.2.1 [33], and alignment files were converted to BAM format using SAMtools v1.18 [34]. Gene-level read counts were obtained using the Rsubread package v2.14.2 [35], and differential gene expression analysis was performed in R using DESeq2 v1.40.2 [36]. Genes with an adjusted *p*-value (Benjamini–Hochberg false discovery rate, FDR) < 0.05 were considered significantly differentially expressed. Functional annotation was performed using the Homo.sapiens v1.3.1 R package v4.3.x (R Foundation for Statistical Computing, Vienna, Austria) and the Ensembl database. Gene set enrichment and pathway analysis were conducted using the fgsea package v1.26.0 based on significantly differentially expressed genes.

### 4.13. Statistical Analysis

Statistical analysis was performed using GraphPad Prism software ver. 10. All experiments were performed at least three times independently unless stated otherwise. Data are presented as mean value (M) ± standard deviation (SD). To determine the statistical significance of the differences an unpaired two-sample Student’s *t*-test was used. To compare the three samples, univariate analysis of variance (ANOVA) followed by the application of Dunnett’s test for comparison with the control sample was used. Regression analysis, curve fitting, and comparison of the obtained data were carried out for proliferation experiments and the assessment of tumor cell sensitivity to paclitaxel. The results were considered statistically significant at *p* < 0.05.

## 5. Conclusions

In summary, cytotoxic macrophages were found to exert a strong selective effect on prostate cancer cells, leading to the emergence of more aggressive tumor variants. The resulting phenotype was associated with broad adaptive changes at both the cellular and molecular levels. These observations indicate that inflammatory pressure within the tumor microenvironment may contribute to tumor evolution in an unexpected way. This supports re-evaluation of the functional role of M1-like macrophages in cancer progression.

## Figures and Tables

**Table 1:** KEGG pathway enrichment analysis of significantly differentially expressed genes in PC3-res cells compared with control PC3 cells. NES—normalized enrichment score.

Pathway	Genes	Direction	NES	q-Value (FDR)
NOD-like receptor signaling pathway	121	Up	2.1688	0.0023
Spliceosome	123	Up	2.1191	0.0023
Ribosome biogenesis in eukaryotes	69	Up	2.0159	0.0082
Cytosolic DNA-sensing pathway	40	Up	2.0021	0.027
Proteasome	38	Up	1.9967	0.03
RNA transport	141	Up	1.9711	0.0023
Metabolism of xenobiotics by cytochrome P450	27	Down	−2.021	0.0075
ECM–receptor interaction	50	Down	−1.9486	0.0075
PPAR signaling pathway	42	Down	−1.9228	0.01
Chemical carcinogenesis	29	Down	−1.8974	0.02
AMPK signaling pathway	94	Down	−1.8543	0.0075
Steroid hormone biosynthesis	21	Down	−1.8522	0.039
Insulin resistance	80	Down	−1.8044	0.01

**Table 2:** Functionally categorized differentially expressed genes identified by RNA-seq analysis of macrophage-selected prostate cancer cells.

Gene	log_2_ Fold Change	Adj. *p*-Value	Biological Role
Innate immune sensing/interferon signaling			
*IFI16*	7.92823819	7.66878 × 10^−70^	Cytosolic DNA sensing; interferon response
*TLR4*	2.208023265	2.5316 × 10^−13^	Innate immune receptor; inflammatory signaling
*GBP4*	2.548542178	4.21938 × 10^−6^	Interferon-stimulated effector protein
*TRIM22*	2.929487927	0.000155001	Interferon-induced regulator; stress adaptation
Immune interaction/antigen presentation			
*CD74*	5.607382165	0.000214022	MHC class II chaperone; antigen presentation
*HLA-DQB1*	4.687751132	3.7222 × 10^−7^	MHC class II molecule; immune interaction
*BST2*	3.85236346	2.93296 × 10^−22^	Interferon-inducible membrane protein
*IL7*	2.958775568	0.008594864	Cytokine signaling; immune modulation
Plasticity/microenvironment adaptation			
*CDH11*	2.325837269	7.08477 × 10^−9^	Cell adhesion; stromal interaction
*PTN*	2.261558412	2.53 × 10^−12^	Growth factor; tumor niche signaling
*NGFR*	2.351533205	0.001905213	Stress/adaptation receptor; cellular plasticity
*FAT3*	3.664431432	1.60941 × 10^−9^	Atypical cadherin; tissue architecture remodeling
*LAPTM5*	2.651972776	3.06645 × 10^−10^	Lysosomal protein; proteostasis and survival
Metabolic & differentiation programs			
*UGT1A6*	−2.298137402	0.004245511	Xenobiotic metabolism enzyme
*SCD*	−2.307636181	1.03109 × 10^−7^	Lipid metabolism regulation
*ASNS*	−2.462321302	0.025469945	Amino acid metabolism
*TFF1*	−2.692525716	3.58607 × 10^−6^	Epithelial differentiation marker
*CASP14*	−2.210385995	0.019900273	Differentiation-associated caspase

**Table 3:** PCR primers sequences.

Gene	Forward Primer	Reverse Primer	Annealing Temp, °C
*GAPDH*	5′-TCGGAGTCAACGGATTTGGT	5′-TCCCGTTCTCAGCCTTGACG	60
*CDH11*	5′-CAGCAGAAATCCACAATCGG	5′-GATCACTCTCACAGATGAAACC	57
*FLOT-1*	5′-AAGACTAAGCAGCAGATTGAG	5′-GCATAATTAGTTGGGACTTCTCTG	57
*HPGD*	5′-TGAGTAAGCAAAATGGAGGTGAAG	5′-GCTGTGCAACGGGCATG	60
*IGFBP3*	5′-GTGTCTGATCCCAAGTTCCA	5′-GCAGGGACCATATTCTGTCTC	58
*PALMD*	5′-TCAGGCTTGAGAAAGAGATCC	5′-GTCCTCAATTGACTCTTCTGCT	58
*PARP8*	5′-GTAGGTCTGCGTTGGAATCTC	5′-GATATGGTGCTTGTGTCATTTCTC	58

## Data Availability

The data presented in this study are available on request from the corresponding author due to the Institution Policy.
